# Recognition of Activities of Daily Living Based on Environmental Analyses Using Audio Fingerprinting Techniques: A Systematic Review

**DOI:** 10.3390/s18010160

**Published:** 2018-01-09

**Authors:** Ivan Miguel Pires, Rui Santos, Nuno Pombo, Nuno M. Garcia, Francisco Flórez-Revuelta, Susanna Spinsante, Rossitza Goleva, Eftim Zdravevski

**Affiliations:** 1 Instituto de Telecomunicações, Universidade da Beira Interior, 6201-001 Covilhã, Portugal; impires@it.ubi.pt (I.M.P.); rui_17_santos@hotmail.com (R.S.); ngpombo@ubi.pt (N.P.); 2 Altranportugal, 1990-096 Lisbon, Portugal; 3 ALLab—Assisted Living Computing and Telecommunications Laboratory, Computing Science Department, Universidade da Beira Interior, 6201-001 Covilhã, Portugal; 4 ECATI, Universidade Lusófona de Humanidades e Tecnologias, 1749-024 Lisbon, Portugal; 5 Department of Computer Technology, Universidad de Alicante, 03690 Sant Vicent del Raspeig, Alicante, Spain; francisco.florez@ua.es; 6 Department of Information Engineering, Marche Polytechnic University, 60121 Ancona, Italy; s.spinsante@univpm.it; 7 Department of Informatics, New Bulgarian University, 1618 g.k. Ovcha kupel 2 Sofia, Bulgaria; rgoleva@gmail.com; 8 Faculty of Computer Science and Engineering, University Ss Cyril and Methodius, 1000 Skopje, Macedonia; eftim.zdravevski@finki.ukim.mk

**Keywords:** acoustic sensors, fingerprint recognition, data processing, artificial intelligence, mobile computing, signal processing algorithms, systematic review, Activities of Daily Living (ADL)

## Abstract

An increase in the accuracy of identification of Activities of Daily Living (ADL) is very important for different goals of Enhanced Living Environments and for Ambient Assisted Living (AAL) tasks. This increase may be achieved through identification of the surrounding environment. Although this is usually used to identify the location, ADL recognition can be improved with the identification of the sound in that particular environment. This paper reviews audio fingerprinting techniques that can be used with the acoustic data acquired from mobile devices. A comprehensive literature search was conducted in order to identify relevant English language works aimed at the identification of the environment of ADLs using data acquired with mobile devices, published between 2002 and 2017. In total, 40 studies were analyzed and selected from 115 citations. The results highlight several audio fingerprinting techniques, including Modified discrete cosine transform (MDCT), Mel-frequency cepstrum coefficients (MFCC), Principal Component Analysis (PCA), Fast Fourier Transform (FFT), Gaussian mixture models (GMM), likelihood estimation, logarithmic moduled complex lapped transform (LMCLT), support vector machine (SVM), constant Q transform (CQT), symmetric pairwise boosting (SPB), Philips robust hash (PRH), linear discriminant analysis (LDA) and discrete cosine transform (DCT).

## 1. Introduction

The identification of Activities of Daily Living (ADL) [1] is of utmost importance to build Enhanced Living Environment and Ambient Assisted Living solutions [2,3], or to allow the development of Personal Digital Life Coaching systems [4]. To achieve this, several authors have proposed the development of solutions based on mobile devices (e.g., smartphones) [5,6,7,8] for several reasons, the most prominent being the adoption ratios of these devices, its increasing computing power and memory, and the fact that these devices already come equipped with a plethora of sensors that can be used to sense and feed data to ADL identification systems.

Despite the increasing complexity of ADL identification systems, the recognition of the surrounding environment is limited because of the restrictions of some location sensors. For instance, Global Positioning System (GPS) sensors, can only be reliably and accurately used in outdoor scenarios. Likewise, proximity sensors, radar sensors, Passive Infra-Red (PIR) sensors and alike require significant installation effort, thus are not widely used in real scenarios which require ADL identification. As proposed in previous works [9,10,11], an ADL identification framework should also be able to integrate data from the sound of the environment into the ADL identification module in order to allow the system to sense the environmental sounds, to determine the type of environment, and to increase the accuracy of the overall ADL identification solution.

Most mobile devices are equipped with a microphone that can capture an acoustic signal. This signal can be processed using audio fingerprinting techniques, allowing the system to find a match between the collected signal and a database of well-known audio fingerprints. This might facilitate an increase in the accuracy of recognition of the environment where ADLs are performed.

Several methods may be used to carry out audio fingerprinting, performing the pre-processing of the acoustic data (e.g., Fast Fourier Transform (FFT)), extracting relevant features, and after that, obtaining a classification or recognition (e.g., Support Vector Machine (SVM)).

This review summarizes the existing methods in the literature related to audio fingerprinting techniques for the application in a system that uses mobile technology for the recognition of the environment. While acknowledging that the methods here presented are very diverse and have been tested with different data sets and different feature extraction techniques, in order to estimate which method may provide better results in a mobile computational device, this paper also presents a comparison between the different methods and features.

The remainder of this paper is organized as follows: Section 2 presents the methodology for this review; the methods discovered in the literature are presented in Section 3; Section 4 discusses different methods, and finally, Section 5 present conclusions of this review.

## 2. Methodology

### 2.1. Research Questions

The primary questions of this review were as follows: (RQ1) What is audio fingerprinting? (RQ2) Which audio fingerprinting techniques are useful to identify the environment of daily activities? (RQ3) Which are the audio fingerprinting techniques feasible for their use in mobile devices?

### 2.2. Inclusion Criteria

Studies assessing ADLs using audio fingerprinting techniques were included in this review if they met the following criteria: (1) audio fingerprinting techniques adapted to mobile devices; (2) audio fingerprinting techniques used for the detection of the environment of ADL; (3) using mobile devices; (4) the accuracies of the audio fingerprinting techniques presented are reported; (5) were published between 2002 and 2017; and (6) were written in English.

### 2.3. Search Strategy

The team searched for studies meeting the inclusion criteria in the following electronic databases: IEEE Xplore, and ACM Digital Library. Every study was independently evaluated by eight reviewers (IP, RS, NP, NG FR, SP, RG and EZ), and its suitability was determined with the agreement of all parties. The studies were examined to identify the characteristics of audio fingerprint and its suitability for application with mobile devices for the identification of ADL.

### 2.4. Extraction of Study Characteristics

The following data was extracted from the studies and tabulated (see Table 1 and Table 2): year of publication, population for the application of the algorithm, purpose of the study, devices used, and study outcomes of the algorithm for audio fingerprinting. For all cited studies in Table 1 and Table 2, the experiments were conducted in laboratory settings. We additionally verified whether the raw data and source code are available, either publically or per request, by emailing the corresponding author of each study.

## 3. Results

**Figure 1:**
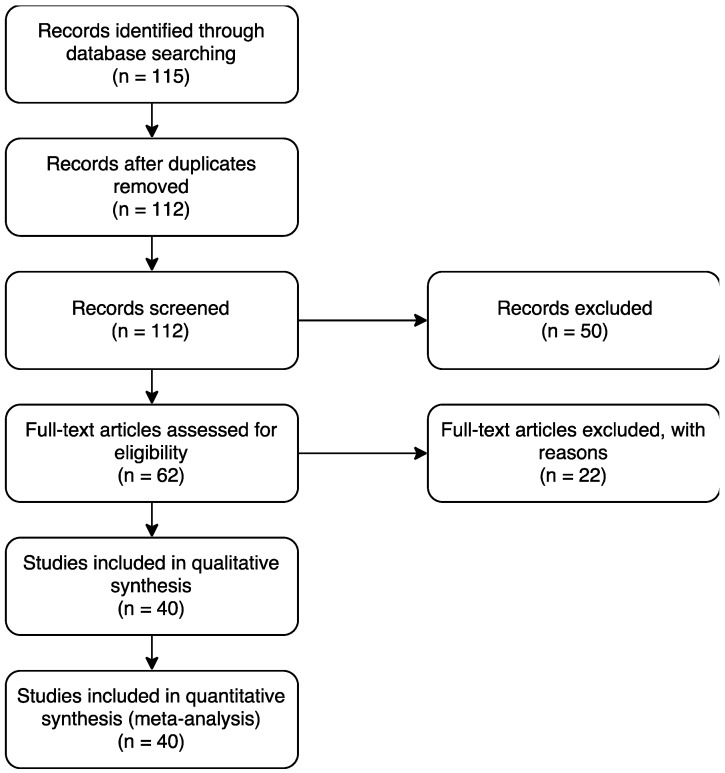
Flow diagram of identification and inclusion of papers.

As illustrated in Figure 1, our review identified 115 papers that included three duplicates, which were removed. The remaining 112 works were evaluated in terms of title, abstract, and keywords, resulting in the exclusion of 50 citations. Full text evaluation of the remaining 62 papers resulted in the exclusion of 22 papers that did not match the defined criteria. The remaining 40 papers were included in the qualitative synthesis and the quantitative synthesis. In summary, our review examined 40 papers.

We suggest that interested readers refer to the original cited works to find relevant information about the details of the methods analyzed in this review. Table 1 shows the year of publication, population, purpose of the study, devices, and settings of the selected papers. Table 2 shows study aims and results. As shown in Table 1, all studies have been performed in controlled environments (laboratory). The major part of the studies was performed between 2011 and 2016 with a total of 29 studies (73%), where five studies were in 2011 (13%), five studies in 2012 (13%), four studies in 2013 (10%), eight studies in 2014 (20%), six studies in 2015 (15%), and one study in 2016 (3%). Some studies indicate the devices used: eight studies used computer microphones (23%), 10 studies used mobile devices (25%), and two studies used a television (5%).

### Methods for Audio Fingerprinting

In [12], the authors created a system that implements the framing, Fast Fourier Transform (FFT), calculation of the spectrum modules, extraction of two kinds of audio fingerprinting, and two level search of two kinds of audio fingerprinting. The two kinds were extracted calculating the sum of the spectrum modulus of every frame, getting the sum of global spectrum modulus in two stages. The authors reported that, when the signal noise rate (SNR) is 10 dB, the two level algorithm, with parallel processing, reports a precision of 100% [12].

In [14], several MP3 features were extracted, such as the Single local description, the Multiple local description, the Modified discrete cosine transform (MDCT), the Mel-frequency cepstrum coefficients (MFCC), the MPEG-7 descriptors, and the chroma vectors, using the Principal Component Analysis (PCA) technique to reduce the dimensionality and QUery Context (QUC)-tree to search for songs. The tests of the methods described in [14] were performed with 10,000 MP3 fragments, reporting a maximum average error equals to 4.26%, which represents an accuracy around 96%. In [13], the same authors extracted the same features and used the same techniques, but they also used the MP3 signatures for the implementation of the audio fingerprinting method, performing tests with 100,000 MP3 fragments, which reported the errors and accuracies obtained are equals to the reported in [14].

Tsai et al. [15] presented a method to calculate audio fingerprints with 6 steps, namely compute spectrogram, collect context frames, apply eigenfilters, compute deltas, apply threshold, and bit packing. The authors reported that the developed method is more robust than the three other fingerprints (e.g., Shazam, Masked Audio Spectral Keypoints (MASK), and Panako), achieving an accuracy of 99.2% [15].

Another audio feature named local energy centroid (LEC) is used in [37] to obtain a representation of audio signals in noisy condition. The method for audio fingerprinting has several steps. First, the audio is downsampled to 8 kHz and segmented into frames, and then FFT is employed to obtain the spectrum. Later, the spectrum is optimized by applying weighted window functions with different size. Then, the LEC is saved and the amplitude components are removed, obtaining an audio spectrum that can be represented by sparse LEC set of coordinates [37]. The authors reported that the method is robust to different noise conditions, and when the linear speed is not changed, the audio fingerprint method based on LEC reports an accuracy of 100% [37].

In [36], the authors proposed an audio fingerprinting algorithm that starts with the application of low-pass filter to the audio signal and resampling to eliminate the high-frequency noise and other audio components that are perceptually insignificant for human auditory system. Afterwards, the audio is framed and weighted by Window function, and the FFT is applied [36]. Next, the Spectral Subband Centroid (SSC) is calculated and the approach of harmonic enhancement is adopted to estimate the predominant pitch of audio signal [36]. Finally, the normalized SSC is masked by the predominant pitch, and the proposed algorithm is resistant to some kinds of signal degradations in varying degrees, reporting an accuracy between 86% and 93% [36]. The authors of [35] also used the normalized SSC for the creation of an audio fingerprinting algorithm. The algorithm is structured in several phases, such as: pre-processing, framing, implementing the FFT to transform audio signals from time to frequency domain, implementing the dynamic subband locating, and applying the normalized SSC, obtaining, at the end, the audio fingerprint [35]. With the fingerprints created, the authors reported an accuracy up to 80% in normal conditions [35]. The authors of [49] also proposed an audio fingerprinting algorithm using SSC, starting with the conversion to mono and downsampling the audio to 11,025 Hz. After the downsampling, the audio signal is windowed by Hamming window (typically 371.5 ms) with 50% overlap and transformed into the frequency domain using FFT [49]. Afterwards, the audio spectrum is divided into 16 critical bands, and the frequency centroids of the 16 critical bands are used as a fingerprint of the audio frame [49], reporting an accuracy around 60% with MP3 and Random start, and an accuracy around 100% with Equalization.

Another algorithm is presented in [50] that consists of the modification of an existing algorithm named Streaming Audio Fingerprinting (SAF), which apply the framing and the FFT, create energy 33 bands, and then, apply a filter and a threshold. The modification of the algorithm consists of increasing the number of the energy bands, and three new steps between the creation of energy bands and the application of a filter and threshold: auto-correction, filter and the creation of a subsample [50]. The authors reported that the algorithm is robust in case of linear speed changes [50].

In [28], the audio fingerprinting methods proposed has several steps, these are framing, application of FFT or quadratically interpolated FFT (QIFFT), time averaging, peak detection, quadratic interpolation, sinusoidal quantification, frequency-axial discretization, and time-axial warping. A fingerprint that represents the distribution of pseudosinusoidal components in the time-frequency domain is generated, showing results with an accuracy around 96% and precision of 100% [28].

In [51], the authors proposed a new fingerprint algorithm based on the streaming approach, where the audio signal is segmented into overlapping frames, the FFT should be applied, and after that, the Human Auditory System (HAS) is used, reporting an accuracy of 100% for the recognition of pop-music.

In [45] is proposed a system for audio fingerprinting that starts with preprocessing and framing of the audio signal. Afterwards, a general feature extraction paradigm, extended with a descriptor based on structural similarity analysis with MPEG-7 Audio Spectrum Flatness (ASF), is applied to the signal [45]. The last step, before the fingerprint construction, consists of the structural analysis that results only the feature vector of the expressive audio piece [45]. At the end, the authors reduce the dimension of the ASF feature vector in the fingerprint construction stage based on the MPEG-7 Audio Signature (AS) description scheme [45], reporting an accuracy around 93%.

The authors of [46] proposed an audio fingerprinting scheme with several stages, such as preprocessing, framing, feature extraction, Gaussian mixture models (GMM) modelling, likelihood estimation, and comparison with a fingerprinting database. In the preprocessing stage, the audio signal is converted to a standard format (16-bit, pulse code modulation (PCM)) [46]. In the framing stage, the audio signals are divided into frames of length equals to 23 ms [46]. During feature extraction, the authors used the STFT, extracting several features, such as Shannon entropy, Rényi entropy, Spectral centroid, Spectral bandwidth, Spectral band energy, Spectral flatness measure, Spectral crest factor, and Mel-frequency cepstral coefficients (MFCC) [46]. Afterwards, the GMM models are applied, using the probability density function (PDF), and the Expectation-Maximization (EM) [46]. Among the features used, spectral centroid gives the highest identification rate of 99.2% [46].

The authors of [47] presented an audio fingerprint extraction algorithm, consisting of: downsampling of the input audio content of 3 s to obtain a sampling rate of 5512 Hz; applying the framing division on the downsampled content using Hamming window with an overlap factor of 31/32; computing the forward balanced multiwavelet (BMW) to transform for each audio frame using five decomposition levels; dividing the subbands’ coefficients into 32 different blocks; applying the estimation quantization (EQ) scheme using a neighbouring window of five audio samples; computing the log variances of the magnitudes of the subbands’ coefficients; computing the mean value of all the log variances for each audio frame; and at the end, extracting the sub-hash bit. Authors report that the performance of the algorithm degrades as the compression rate increases.

In [48], the authors proposed an algorithm with two stages named indexing and search. The indexing is based in the construction of zone tables using the Search by Range Reduction (SRR) threshold values [48]. The search is based on the SRR test, calculating the Itakura distance between two fingerprints, and comparing it with values in the zone tables [48]. An accuracy of around 98% is reported.

The authors of [43] proposed an algorithm with training and testing phases. For the training phase, the authors started with the wavelet packet decomposition, and developed a local discriminant bases (LDBs)-based automated multigroup audio classification system, which focuses on identifying discriminatory time-frequency subspaces [43]. The testing phase consists of the construction of a new wavelet tree, feature extraction, and implementation of a linear discriminant analysis (LDA) [43]. The extracted features include MFCC, spectral similarity, timbral texture, band periodicity, linear prediction coefficient derived cepstral coefficients (LPCCs), zero crossing rate, MPEG-7 descriptors, entropy, and octaves [43]. The authors of [43] reported that the average classification accuracy was between 91% and 99% [43].

The authors of [44] presents a video retrieval system (VRS) for Interactive-Television as like internet protocol television (IPTV), which implements an audio fingerprint feature of long-term logarithmic modified discrete cosine transform (DCT) modulation coefficients (LMDCT-MC) for audio indexing and retrieval, and implements two-stage search (TSS) algorithm for fast searching. In the first stage of TSS, candidate video segments are roughly determined with audio index bit vectors (IBV) and then the optimal video clip is obtained by fingerprint bit vectors (FBV). An accuracy of 99.67% is reported in [44].

In [41] an audio fingerprint method using sub-fingerprint masking based on the predominant pitch extraction is proposed. It increases the accuracy of the audio fingerprinting system in a noisy environment dramatically, while requiring much less computing power compared to the expanded hash table lookup method. When applied to an audio signal without noise, the reported accuracy is 97.4%.

The authors of [42] presented a sub-Nyquist audio fingerprinting system for music recognition, which utilizes Compressive Sampling (CS) theory to generate a compact audio fingerprint, and to achieve significant reduction of the dimensionality of the input signal, compared to Nyquist sampling methods [42]. The average accuracy of this method is 93.43% under various distorted environments.

In [38], the authors presented an algorithm based on fingerprinting techniques implemented in a low-cost embedded reconfigurable platform. It utilizes the FFT implementation from the CUFFT library, based on the Fastest Fourier Transform in the West (FFTW) algorithm. This approach normalizes and frames the audio signal, computes the correlation and cross correlation, and applies a derivative of the audio signal. An accuracy of 94% is reported.

The authors of [39] created a fingerprint database of songs and focused on the problem of effective and efficient database search. The authors proposed a new indexing scheme that overcomes the limitations of Haitsma-Kalker’s method and Miller’s k-ary tree method, adopting the inverted file as the underlying index structure and developing the techniques to apply it to the effective and efficient audio fingerprinting problem. An accuracy higher than 97% is reported in [39], which is the performance of the Haitsma-Kalker’s method.

The authors of [40] explored a common audio-fingerprinting approach with the implementation of FFT, and taken into account the noise in the derived fingerprints by employing error correcting codes and applying statistical tests. Testing with several sample windows of Network Time Protocol (NTP)-based synchronization recordings, authors of [40] reported an accuracy between 60% and 70%.

The authors of [31] created a system based on a client-server architecture able to recognize a live television show using audio fingerprinting. To create audio fingerprints, FFT is computed to obtain the power spectrum, which is integrated over a pre-defined set of non-overlapping, logarithmically spaced frequency bins and eventually squared to obtain an energy measure [31]. The likelihood estimation based on the cross-correlation function was used for comparison of the audio fingerprints. An accuracy of around 95% is reported in [31].

The authors of [32] presented an audio fingerprint method named Masked Audio Spectral Keypoints (MASK), which encodes the acoustic information existent in audio documents and discriminates between transformed versions of the same acoustic documents and other unrelated documents. The MASK fingerprint extraction method is composed of several tasks: time-to-frequency transformation, where the input signal is transformed from the time domain to the spectral domain, and transformed into Mel-scale; salient spectral points search; local mask application around each of the salient points; grouping of the different spectrogram values into regions; and the MASK fingerprint encoding and storage. The averaged energy values of each one of these spectrogram regions are compared to construct a fixed length binary descriptor. Authors of [32] report an accuracy around 58%.

In [33], the authors implemented an audio fingerprinting algorithm based on fingerprint extraction and matching search, adapting the well-known Philips’ algorithm. The fingerprint extraction derives and encodes a set of relevant audio features, which need to be invariant to various kinds of signal distortion, including background noise, audio compression, and A/D conversion [33]. The matching search finds the best match between these fingerprints and those stored in the database [33]. The implemented audio fingerprint extraction method uses FFT, and extracts several features, such as: mel-frequency cepstral coefficients (MFCC), spectral centroid or spectral flatness [33]. The audio fingerprinting method reports an accuracy of 95% and a precision of 100% [33].

The authors of [34] proposed an audio fingerprinting system with several characteristics, including robustness, granularity, and retrieval speed, reporting an accuracy between 88% and 99%. The structure of the audio fingerprinting implemented is the same as all other algorithms presented in [34], applying the FFT and an High-pass filter. The authors used the local maximum chroma energy (LMCE) to extract the perception features of Tempo-Frequency domain [34].

The work presented in [29] reviews the state-of-the-art methods for improving the power consumption and computation speed to make the smartphone implementation. It also proposed the Improved Real-Time TV-channel Recognition (IRTR), which is a fingerprint extraction method aimed at recognizing in real time what people are watching on TV without any active user interaction. The identification using the audio fingerprint is performed using a likelihood estimation [29]. The audio fingerprinting method implements linear transform and feature extraction, with several steps: the audio is recorded and divided into frames with overlap factor; each frame is filtered by means of a Hamming window function; the application of the FFT and the squared modulus; the spectrum is divided into logarithmically spaced frequency bins and the energy is computed for each bin; and the nervy of band of each frame is denoted. An accuracy about 95% is reported in [29].

In [30], an audio fingerprinting algorithm is proposed for efficient retrieval of corresponding or similar items from large audio databases, which improves the of the database search compared to the algorithm used in Haitsma’s method, without impairing the accuracy of the search results. The approach implements the FFT, the extraction of candidate songs via lookup table, the assignment of weights to candidate songs, and the database search [30], while reporting an average accuracy around 81%.

The authors of [21] proposed an audio fingerprinting algorithm, which improves binary audio fingerprint matching performance by utilizing auxiliary information. The proposed matching method is based on Philips robust hash (PRH) for audio signal; Asymmetric Fingerprint Matching for PRH using the Magnitude Information, which consists of Normalization of the Subband-Energy Difference; and Fingerprint Matching Based on the Likelihood Ratio Test [21]. The proposed method yields better performance than the conventional Hamming distance [21].

The authors of [22] proposed an audio fingerprinting constituted by two stages: fingerprinting and matching. The fingerprinting module uses a log-frequency spectrogram based on the Constant Q Transform (CQT), and an adaptive thresholding method based on two-dimensional median filtering [22]. The matching uses the Hamming similarity and the Hough Transform [22]. The reported accuracy is between 61% and 81%.

The authors of [23] presented a method for the construction of audio fingerprints based on: maximization of the mutual information across the distortion channel; using the information bottleneck method to optimize the filters; and quantizers that generate these fingerprints. The method starts with the application of the short time Fourier transform (STFT), and capturing the Spectral Sub-band Centroids (SSC) using 16 bins on the Bark scale. The generated features with [23] result in a maximum accuracy of around 65%.

The authors of [24] implemented an audio fingerprinting algorithm composed by several steps: downsampling to 5 kHz, segmenting frames every 11.6 ms, applying the FFT, calculating the frequency bands energies, and finally, calculating the fingerprints. A recall around 98% is reported.

In [25], the authors presented an audio fingerprinting algorithm based on the compressed-domain spectral entropy as audio features, showing strong robustness against various audio signal distortions such as recompression, noise interference, echo addition, equalization, band-pass filtering, pitch shifting, moderate time-scale modification, among others. The algorithm includes four steps: granule grouping, frequency alignment between long and short windows, coefficients selection and subband division, and MDCT spectral entropy calculation and fingerprint modelling [25]. It reports an accuracy above 90%.

In [26], the authors presented the implementation of an audio fingerprinting system, using graphic processing units (GPUs). The system starts with the extraction of landmarks using FFT, and continues with the landmark extraction, lookup, and analysis. The authors explored the use of one thread for one hash key, and one block for one hash key, reporting an accuracy around 80.96%, when there are 100,000 songs in the database [26].

The authors of [27] proposed a high-performance audio fingerprint extraction method for identifying TV commercial advertisement. The audio fingerprint extraction consists of a salient audio peak pair fingerprints based on constant Q transform (CQT). The algorithm obtains the audio fingerprints through five main steps: preprocessing; application of the CQT; application of the Mean Subtraction of Logarithmic CQT Spectrum; application of the CQT Based Salient Peak Detection using forward and Backward Filtering; and finally, application of the Fingerprint Generation using CQT Peak Pair. The reported recognition accuracy of the method based on CQT, presented in [27], is around 89.8%.

The authors of [16] used a smartphone and create an audio fingerprinting algorithm based on the joint usage of GPS and acoustic fingerprints. The authors created an audio fingerprinting algorithm with noise tolerance, assessing it under several conditions [16]. The algorithm starts with the calculation of the audio sample spectrogram using the STFT, and then calculates audio sample spectrogram using the Hamming window and a high overlap [16]. Next, it takes only the first 40 frequency bins, as most of the useful audio features are in that bandwidth, averaging the logarithmic amplitude in each bin [16]. Afterwards, for each frequency bin, a 16-bit fingerprint is calculated [16]. The 16-bits fingerprint is then stored with the associated frequency and time [16]. For the comparison of the audio fingerprints, the Hamming distances between each fingerprint are calculated, looking for a minimum [16]. An accuracy of around 86% is reported.

In [17], the authors proposed an approach to accelerate fingerprinting techniques by skipping the search for irrelevant sections of the signal and demonstrate its application to the divide and locate (DAL) audio fingerprint method. The method in DAL starts with the extraction of the time-frequency power spectral applied for the signals, normalizing each logarithmic power [17]. Afterwards, the normalized data is decomposed into a number of small time-frequency components of uniform size, and thus, the computational cost and memory usage are reduced in the fingerprint data [17]. The authors verified that with a reduced search threshold, the accuracy of the recognition is around 100% [17].

The authors of [18] created a method to analyze and classify daily activities in personal audio recordings (PARs). The method applies: speech activity detection (SAD), speaker diarization systems, and computing the number of audio speech and lexical features [18]. It uses a TO-Combo-SAD (Threshold Optimized Combo SAD) algorithm for separating speech from noise [18]. The Principal Component Analysis (PCA) is first applied for dimensionality reduction, and then, the remaining features are supplied to a multi-class support vector machine (SVM) with radial basis function (RBF) kernel for model training and evaluation [18]. The authors performed recognition of faculty meeting, research meeting, staff meeting, alone time, and conference call, reporting accuracies between 62.78% and 84.25% [18].

In [19], the authors proposed an audio fingerprinting method, based on landmarks in the audio spectrogram. The algorithm is based on the audio hashing of frequency peaks in the spectrogram [19]. It starts with the application of the FFT, thresholding the data, applying a high pass filter, identifying the local maximums and finding the peaks of the spectrogram [19]. The performance of the algorithm decreases at higher additive noise in comparison with other algorithms [19], reporting an accuracy around 96.71%.

In [20], the authors proposed a robust TV advertisement search based on audio fingerprinting in real environments. This algorithm has several steps, such as preprocessing, logarithmic moduled complex lapped transform (LMCLT), two-are segmentation using adaptive thresholding based on median filtering, detection of prominent LMCLT spectral peaks, and fingerprinting generation using LMCLT peak pair [20]. The method applies adaptive peak-picking thresholding method to extract more salient and distinct peak pairs for comparing the query fingerprint with the original fingerprints, and the authors reported an accuracy of 86.5% [20].

## 4. Discussion

This review confirms the findings of previous studies related to the use of audio fingerprinting techniques for identification of the environment related to the different ADLs. We consider that many of the reviewed works raise important issues regarding the concept of Open Science, including, but not limited to, Reproducibility and Verifiability of the research results, and Comparability of similar research. Many of them were evaluated on unpublished data and did not publish their source code, although when commercial solutions are in perspective, a necessary degree of confidentiality is understandable. Regarding validation and comparability, only six studies used raw data available online or published its research data online. Likewise, only three studies presented some parts of the code used of the experiments. In addition, the studies that used data that is now publicly available, did not publish the research source code, making the validation of the results and further comparative research an impossible task. Therefore, we suggest to the audio fingerprinting community to become better at sharing raw data and algorithms, so as to be able to recreate and evaluate the soundness of previous studies.

Nevertheless, assuming the results of the presented research studies are comparable, Table 3, Table 4 and Table 5 present a summary of the Features and Methods ordered by the number of identified studies that use these features and methods.

Table 3 and Table 4 present the distribution of the extracted features and methods implemented in the analyzed studies, verifying that FFT is one of the most widely used feature extraction method, because it extracts the frequencies from the audio signal, and the other most used features include thresholding, normalized Spectral Subband Centroid (SSC), Mel-frequency cepstrum coefficients (MFCC), maximum, local peaks and landmarks, Shannon entropy, Rényi entropy, MPEG-7 descriptors, Spectral bandwidth, Spectral flatness measure, Modified discrete cosine transform (MDCT), Constant Q Transform (CQT), Short-time Fourier transform (STFT), average, and the maximum and minimum. These features were used in a large part of the analyzed studies [12,14,19,24,26,28,29,30,31,33,34,35,38,49,50,51], and with them, the reported accuracy is greater than 80%, as presented in Table 3.

For Table 3 and Table 4, the accuracies that are equal or higher than 99% are shown in a different background color (yellow).

On the other hand, as verified in Table 4, a large part of the analyzed studies [12,14,19,24,26,28,29,30,31,33,34,35,38,49,50,51] do not mention the name of the method applied, presenting only the features used. Regarding the undifferentiated methods, the most used methods are the two level search algorithm, the likelihood estimation, the Principal Component Analysis (PCA), and the Hamming distances between each fingerprint, reporting accuracies also higher than 80%.

Table 5 presents in a matrix format, the average of the averages of the accuracies in Methods vs. its Features. This table is a mere comparison exercise, as there are not enough studies to sustain a valid analysis of the use of different features with different methods. On the other hand, this table assumes that these results are comparable, and moreover, that any method or algorithm can be used with any set of features, which of course, is a very wide, and possibly not true assumption. Nevertheless, Table 5 shows, in a colored background the match between features and methods. For example, for method SAF (Streaming Audio Fingerprinting) the set of used features are Fast Fourier Transform, Thresholds and Energy bands, whose mean accuracies in the found studies are not higher than 99%. Also, for example for the method GMM (Gaussian Mixture Models Modelling), besides the 4 highlighted features that were used, this method uses additionally 5 other sets of features.

Taking Table 5 into consideration, one can identify Shannon’s Entropy as the feature that is most used in the most accurate number of studies. Arguably, this table may propose new combinations of features and methods that can be used to devise audio-fingerprinting solutions.

For a particular use, the methods to be implemented must be chosen according to their complexity, the computational power of the use case scenario, and to the purpose of its intended use. This review is focused on the use of mobile devices, but only three of the reviewed works argue that they use methods that need low resources (see Table 1). Only 19 studies compared the implemented methods with other methods published in the literature and present their accuracy, claiming an increased accuracy in the recognition of the environment using audio fingerprinting.

According to the results of this review, the use of the mobile devices for the application of audio fingerprinting techniques is limited, because of the restrictions these devices impose, i.e., low power processing and battery capacity. Thus, only 10 of the analyzed studies utilize mobile devices with local processing or server-side processing of the data acquired from the mobile devices. In the case of the server-side processing, the use of the mobile devices implies a constant and stable network connection, which is not a trivial requirement both from technical perspective, but also because of battery life implications. To some extent, the using Fog and Mist Computing paradigms could overcome the challenges of the client-server architectures. The creation of lightweight techniques should be explored, as they could be executed on mobile devices (i.e., edge-nodes). The models could be recalibrated offline on the server occasionally, and then, as pre-trained models to be seamlessly redeployed on mobile devices.

In conclusion, only one of the reviewed studies [38] can achieve reliable performance with reduced computational cost and memory usage. It utilizes the FFT implementation in the CUFFT library, divide and locate (DAL) audio fingerprint method, and sub-fingerprint masking based on the predominant pitch extraction methods. However, other methods could be implemented on mobile devices with some restrictions. Nonetheless, they could be amended to utilize more lightweight implementations of the underlying libraries, or by sacrificing floating point precision, for instance.

## 5. Conclusions

This review identified and described the methodologies used for audio fingerprinting that can be applied to mobile technologies. Forty-seven studies were examined and the main findings are summarized as follows:

- (RQ1) the audio fingerprinting is defined as the ability to recognize the scenario in which a given audio was collected and involved in, based on various methods.
- (RQ2) Several techniques have been applied to implement audio fingerprinting methods, including Fast Fourier Transform (FFT). Support Vector Machine (SVM), QUery Context (QUC)-tree, spectral subband centroid (SSC), Streaming Audio Fingerprinting (SAF), Human Auditory System (HAS), Gaussian mixture models (GMM) modelling, likelihood estimation, linear discriminant analysis (LDA), Compressive Sampling (CS) theory, Philips robust hash (PRH), Asymmetric Fingerprint Matching, and TO-Combo-SAD (Threshold Optimized Combo SAD). These techniques yield high accuracy, and the use of mobile devices does not influence the predictive performance, allowing the use of these techniques anywhere, anytime.
- (RQ3) All of the methods presented in RQ2 can be implemented on mobile devices, but the methods that require lower computational resources are FFT with the CUFFT library, divide and locate (DAL) audio fingerprint method, and sub-fingerprint masking based on the predominant pitch extraction.

In addition, this review highlights the application of audio fingerprinting techniques on mobile or other devices with limited computational and battery resources. Some limitations of this review should be mentioned. First, the authors chose to exclude studies that are not focused on audio fingerprinting techniques. Second, the studies that do not utilize mobile devices have been excluded. These exclusions were performed with the analysis of the abstract and then, the full text of the papers. Finally, only English-language publications were included.

Based on the analysis, we conclude that the most used methods are undifferentiated methods, two level search algorithms, likelihood estimation, Principal Component Analysis (PCA), and Hamming distances between each fingerprint. The conclusion is that the use of statistical methods reports results with an accuracy higher than 80%. Furthermore, the most used features are Fast Fourier Transform (FFT), Thresholding, normalized spectral subband centroid (SSC), Mel-frequency cepstrum coefficients (MFCC), maximum, local peaks and landmarks, Shannon entropy, Rényi entropy, MPEG-7 descriptors, Spectral bandwidth, Spectral flatness measure. Modified discrete cosine transform (MDCT), Constant Q Transform (CQT), Short-time Fourier transform (STFT), average, and minimum, which also result in accuracies greater than 80%.

As future work, the extraction of features based on audio fingerprinting will be implemented in order to develop a system for the recognition of ADLs and their environments, presented in [9,10,11]. As presented in Table 3, the accuracy is always higher than 80%. Then, we should consider the most used features, including FFT, MFCC, average, maximum, and minimum, in order to better handle the recognition of the environment. The implementation of this framework is part of the development of a personal digital life coach [4].

## Figures and Tables

**Table 1:** Study Analysis.

Paper	Year of Publication	Population	Purpose of the Study	Devices	Raw Data Available	Source Code Available
			ACM			
Sui et al. [12]	2014	2500 pieces of 8 s advertisement audios, and randomly select 200 pieces of audio in the existing database and 50 pieces of other irrelevant audio as test audio	To search for audio in the database by the content rather than by name	Mobile Phone (Android)	No	No
Liu [13]	2012	100,000 MP3 fragments	To create an MP3 sniffer system that includes audio fingerprinting	Not mentioned	Yes	Only for feature extraction
Liu et al. [14]	2011	10,000 MP3 fragments	Proposes an MP3 fingerprint system for the recognition of several clips	Not mentioned	The same data as [13]	The same source code as [13]
			IEEE			
Tsai et al. [15]	2016	Multi-channel audio recordings of 75 real research group meetings, approximately 72 h of meetings in total	Proposes an adaptive audio fingerprint based on spectrotemporal eigenfilters	Mobile phones, tablets or laptop computers	Yes	No
Casagranda et al. [16]	2015	1024 samples	Proposes an audio fingerprinting method that uses GPS and acoustic fingerprints	Smartphone	No	No
Nagano et al. [17]	2015	Approximately 1,518,177 min (25,303 h) of songs	Proposes a method to accelerate audio fingerprinting techniques by skipping the search for irrelevant signal sections	Not mentioned	Yes	No
Ziaei et al. [18]	2015	1062 10 s clips	Proposes a method to analyze and classify daily activities in personal audio recordings	Not mentioned	Yes	No
George et al. [19]	2015	1500 audio files	Proposes an audio fingerprinting method based on landmarks in the audio spectrogram	Computer	No	No
Kim et al. [20]	2015	6000 television advertisements with a total time of 1110 h	Proposes a television advertisement search based on audio fingerprinting in real environments	Television	No	No
Seo [21]	2014	1000 songs with classic, jazz, pop, rock, and hip-hop	Proposes a binary audio fingerprint matching, using auxiliary information	Not mentioned	No	No
Rafii et al. [22]	2014	Several songs with a duration between 6 and 9 s	Proposes an audio fingerprinting method for recognition of some clips	Computer and Smartphone	No	No
Naini et al. [23]	2014	1000 songs	Proposes an audio fingerprinting method based on maximization of the mutual information across the distortion channel	Not mentioned	No	No
Yang et al. [24]	2014	200,000 songs	Proposes a music identification system based on space-saving audio fingerprints	Not mentioned	No	No
Yin et al. [25]	2014	958 randomly chosen query excerpts	Proposes an audio fingerprinting algorithm that uses compressed-domain spectral entropy	Not mentioned	No	No
Wang et al. [26]	2014	100,000 songs	Proposes an audio fingerprinting method that uses GPUs	Not mentioned	No	No
Lee et al. [27]	2014	3000 TV advertisements	Proposes a high-performance audio fingerprint extraction method for identifying Television commercial advertisement	Television	No	No
Shibuya et al. [28]	2013	1374 television programs (792 h in total)	Proposes a method of identifying media content from an audio signal recorded in reverberant and noisy environments using a mobile device	Smartphone, tablet, notebook, desktop, or another mobile device	No	No
Bisio et al. [29]	2013	20 sounds	Proposes the Improved Real-Time TV-channel Recognition (IRTR) method	Smartphone	No	No
Lee et al. [30]	2013	1000 songs as positive samples and 999 songs as negatives	Proposes a method that speeds up the search process, reducing the number of database accesses	Not mentioned	No	No
Bisio et al. [31]	2012	100,000 songs	Proposes an audio fingerprint algorithm adapted to mobile devices	Smartphone	No	No
Anguera et al. [32]	2012	Several datasets	Proposes an audio fingerprinting algorithm that encodes the local spectral energies around salient points selected among the main spectral peaks in a given signal	Not mentioned	No	No
Duong et al. [33]	2012	300 real-world recordings in a living room	Proposes an audio fingerprinting method that combines the Fingerprinting technique with Generalized cross correlation	iPad	No	No
Wang et al. [34]	2012	20 music clips with 5 s	Proposes an audio fingerprinting algorithm for recognition of some clips	Not mentioned	No	No
Xiong et al. [35]	2012	835 popular songs	Proposes an audio fingerprinting algorithm based on dynamic subband locating and normalized spectral subband centroid (SSC)	Not mentioned	No	No
Deng et al. [36]	2011	100 audio files	Proposes an audio fingerprinting algorithm based on harmonic enhancement and SSC of audio signal	Not mentioned	No	No
Pan et al. [37]	2011	62-h audio database of 1000 tracks	Proposes an audio feature in spectrum, local energy centroid, for audio fingerprinting	Not mentioned	No	No
Martinez et al. [38]	2011	3600 s of several real-time tests	Presents an audio fingerprinting method with a low-cost embedded reconfigurable platform	Computer	No	No
Cha [39]	2011	1000 songs	Proposes an indexing scheme and a search algorithm based on the index	Computer	No	Only pseudo-code for fingerprint matching
Schurmann et al. [40]	2011	7500 experiments	Proposes an audio fingerprinting method for the recognition of some clips	Computer	No	No
Son et al. [41]	2010	500 popular songs	Proposes an audio fingerprinting method using sub-fingerprint masking based on the predominant pitch extraction	Mobile devices	Yes	No
Chang et al. [42]	2010	17,208 audio clips	Presents a sub-Nyquist audio fingerprinting system for music recognition, which utilizes Compressive Sampling (CS) theory	Not mentioned	No	No
Umapathy et al. [43]	2007	213 audio signals	Proposes an audio feature extraction and a multi-group classification using the local discriminant bases (LDB) technique	Not mentioned	No	No
Kim et al. [44]	2007	100 Korean broadcast TV programs	Proposes an audio fingerprinting method for identification of bookmarked audio segments	Computer	No	No
Sert et al. [45]	2006	approximately 45 min of pop, rock, and country songs	Proposes an audio fingerprinting method from the most representative section of an audio clip	Not mentioned	No	No
Ramalingam et al. [46]	2006	250 audio files	Proposes and audio fingerprinting method using several features	Not mentioned	No	No
Ghouti et al. [47]	2006	Two audio contents perceptually similar	Proposes an audio fingerprinting algorithm that uses balanced multiwavelets (BMW)	Not mentioned	No	No
Cook et al. [48]	2006	7,106,069 fingerprints	Proposes an audio fingerprinting algorithm for the fast indexing and searching of a metadata database	PDA or computer	Yes	No
Seo et al. [49]	2005	8000 classic, jazz, pop, rock, and hip-hop songs	Proposes an audio fingerprinting method based on normalized SSC	Not mentioned	No	No
Haitsma et al. [50]	2003	256 sub-fingerprints	Proposes to solve larger speed changes by storing the fingerprint at multiple speeds in the database or extracting the fingerprint query at multiple speeds and then to perform multiple queries on the database	Not mentioned	No	No
Haitsma et al. [51]	2002	256 sub-fingerprints	Proposes an audio fingerprinting system for recognition of some clips	Not mentioned	No	No

**Table 2:** Study summaries.

Paper	Outcomes
	**ACM**
Sui et al. [12]	The authors propose a two-level audio fingerprint retrieval algorithm to satisfy the demand of accurate and efficient search for advertisement audio. Based on clips with 8 s of advertisements, the authors build a database with 2500 audio fingerprints. The results show that the algorithm implemented with parallel processing yields a precision of 100%.
Liu [13]	The authors create an MP3 sniffer system and test it with multi-resolution local descriptions. The system has a database of 100,000 MP3 tones and authors report that the system has high performance, because 100 queries for identifying unknown MP3 tones took less than 2 s to be processed
Liu et al. [14]	The authors describe an MP3 fingerprinting system that compares the normalized distance between two MP3 fingerprints to detect a false identification. The authors identify the possible features of the song and build a large database. For the identification, the authors test the near neighbor searching schemes and compare with the indexing scheme, which utilizes the PCA technique, the QUery Context (QUC)-tree, and the MP3 signatures. The conclusions show that the system has a maximum average error equals to 4.26%.
	**IEEE**
Tsai et al. [15]	The authors propose a method for aligning a set of overlapping meeting recordings, which uses an audio fingerprint representation based on spectrotemporal eigenfilters that are learned on-the-fly in an unsupervised manner. The proposed method is able to achieve more than 99% alignment accuracy at a reasonable error tolerance of 0.1 s.
Casagranda et al. [16]	The authors propose an audio fingerprinting algorithm based on the spectral features of the audio samples. The authors reported that the algorithm is noise tolerant, which is a key feature for audio based group detection.
Nagano et al. [17]	The authors propose an approach to accelerate fingerprinting techniques and apply it to the divide-and-locate (DAL) method. The reported results show that DAL3 can reduce the computational cost of DAL to approximately 25%.
Ziaei et al. [18]	The authors propose a method to analyze and classify daily activities in personal audio recordings (PARs), which uses speech activity detection (SAD), speaker diarization, and a number of audio, speech and lexical features to characterize events in daily audio streams. The reported overall accuracy of the method is approximately 82%.
George et al. [19]	The authors propose an audio fingerprinting method that is tolerant to time-stretching and is scalable. The proposed method uses three peaks in the time slice, unlike Shazam, which uses only one. The additive noise deteriorates the lowest frequency bin, decreasing the performance of the algorithm at higher additive noise, compared to other algorithms.
Kim et al. [20]	The authors propose a Television advertisement search based on audio peak-pair hashing method. The reported results show that the proposed method has respectable results compared to other methods.
Seo [21]	The authors propose an asymmetric fingerprint matching method which utilizes an auxiliary information obtained while extracting fingerprints from the input unknown audio. The experiments carried out with one thousand songs against various distortions compare the performance of the asymmetric matching with the conventional Hamming distance. Reported results suggest that the proposed method has better performance than the conventional Hamming distance.
Rafii et al. [22]	The authors propose an audio fingerprinting system with two stages: fingerprinting and matching. The system uses CQT and a threshold method for fingerprinting stage, and the Hamming similarity and the Hough Transform for the matching stage, reporting an accuracy between 61% and 81%.
Naini et al. [23]	The authors present a method for designing fingerprints that maximizes a mutual information metric, using a greedy optimization method that relies on the information bottleneck (IB) method. The results report a maximum accuracy around 65% in the recognition.
Yang et al. [24]	The authors propose an efficient music identification system that utilizes a kind of space-saving audio fingerprints. The experiments were conducted on a database of 200,000 songs and a query set of 20,000 clips compressed in MP3 format with different bit rates. The author’s report that compared to other methods, this method reduces the memory consumption and keeps the recall rate at approximately 98%.
Yin et al. [25]	The authors propose a compressed-domain audio fingerprinting algorithm for MP3 music identification in the Internet of Things. The algorithm achieves promising results on robustness and retrieval precision rates under various time-frequency audio signal distortions including the challenging pitch shifting and time-scale modification.
Wang et al. [26]	The authors propose parallelized schemes for audio fingerprinting over GPU. In the experiments, the speedup factors of the landmark lookup and landmark analysis are verified and the reported overall response time has been reduced.
Lee et al. [27]	The authors propose a salient audio peak pair fingerprint extraction based on CQT. The reported results show that the proposed method has better results compared to other methods, and is suitable for many practical portable consumer devices.
Shibuya et al. [28]	The authors develop a method that uses the quadratically interpolated FFT (QIFFT) for the audio fingerprint generation in order to identify media content from an audio signal recorded in a reverberant or noisy environment with an accuracy around 96%.
Bisio et al. [29]	The authors present an improvement of the parameter configuration used by the Philips audio fingerprint computation algorithm in order to reduce the computational load and consequent energy consumption in the smartphone client. The results show a significant reduction of computational time and power consumption of more than 90% with a limited decrease in recognition performance.
Lee et al. [30]	The authors propose an audio fingerprint search algorithm for music retrieval from large audio databases. The results of the proposed method achieve 80–99% search accuracy for input audio samples of 2–3 s with signal-to-noise ratio (SNR) of 10 dB or above.
Bisio et al. [31]	The authors present an optimization of the Philips Robust Hash audio fingerprint computation algorithm, in order to adapt it to run on a smartphone device. In the experiments, the authors report that the proposed algorithm has an accuracy of 95%.
Anguera et al. [32]	The authors present a novel local audio fingerprint called Masked Audio Spectral Keypoints (MASK) that is able to encode, with few bits, the audio information of any kind in an audio document. MASK fingerprints encode the local energy distribution around salient spectral points by using a compact binary vector. The authors report an accuracy around 58%.
Duong et al. [33]	The authors presented a new approach based on audio fingerprinting techniques. The results of this study indicate that a high level of synchronization accuracy can be achieved for a recording period as short as one second.
Wang et al. [34]	The authors present an audio fingerprinting algorithm, where the audio fingerprints are produced based on 2-Dimagel, reporting an accuracy between 88% and 99%.
Xiong et al. [35]	The authors propose an improved audio fingerprinting algorithm based on dynamic subband locating and normalized Spectral Subband Centroid (SSC). The authors claim that the algorithm can recognize unknown audio clips correctly, even in the presence of severe noise and distortion.
Deng et al. [36]	The authors propose an audio fingerprinting algorithm based on harmonic enhancement and Spectral Subband Centroid (SSC). The authors build a database with 100 audio files, and also implement several techniques to reduce the noise and other degradations, proving the reliability of the method when severe channel distortion is present. The results report an accuracy between 86% and 93%.
Pan et al. [37]	The authors propose a method for fingerprinting generation using the local energy centroid (LEC) as a feature. They report that the method is robust to different noise conditions and, when the linear speed is not changed, the audio fingerprint method based on LEC obtains an accuracy of 100%, reporting better results than Shazam’s fingerprinting.
Martinez et al. [38]	The authors present a music information retrieval algorithm based on audio fingerprinting techniques. The size of frame windows influences the performance of the algorithm, e.g., the best size of the frame window for shorts audio tracks is between 32 ms to 64 ms, and the best size of the frame window for audio tracks is 128 ms.
Cha [39]	The author proposes an indexing scheme for large audio fingerprint databases. The method shows a higher performance than the Haitsma-Kalker method with respect to accuracy and speed.
Schurmann et al. [40]	The authors propose a fuzzy-cryptography scheme that is adaptable in its noise tolerance through the parameters of the error correcting code used and the audio sample length. In a laboratory environment, the authors utilized sets of recordings for five situations at three loudness levels and four relative positions of microphones and audio source. The authors derive the expected Hamming distance among audio fingerprints through 7500 experiments. The fraction of identical bits is above 0.75 for fingerprints from the same audio context, and below 0.55 otherwise.
Son et al. [41]	The authors present an audio fingerprinting algorithm to recognize songs in real noisy environments, which outperforms the original Philips algorithm in recognizing polyphonic music in real similar environments.
Chang et al. [42]	The authors introduce the Compressive Sampling (CS) theory to the audio fingerprinting system for music recognition, by proposing a CS-based sub-Nyquist audio fingerprinting system. Authors claim that this system achieves an accuracy of 93.43% in reducing the sampling rate and in the extraction of musical features.
Umapathy et al. [43]	The authors present a novel local discriminant bases (LDB)-based audio classification scheme covering a wide range of audio signals. After the experiments, the obtained results suggest significant potential for LDB-based audio classification in auditory scene analysis or environment detection.
Kim et al. [44]	The authors develop a system that retrieves desired bookmarked video segments using audio fingerprint techniques based on the logarithmic modified Discrete Cosine Transform (DCT) modulation coefficients (LMDCT-MC) feature and two-stage bit vector searching method. The author’s state that the search accuracy obtained is 99.67%.
Sert et al. [45]	The authors propose an audio fingerprinting model based on the Audio Spectrum Flatness (ASF) and Mel Frequency Cepstral Coefficients (MFCC) features, reporting and accuracy of 93% and 91%, respectively.
Ramalingam et al. [46]	The authors propose a method to create audio fingerprints by Gaussian Mixtures using features extracted from the short-time Fourier transform (STFT) of the signal. The experiments were performed on a database of 250 audio files, obtaining the highest identification rate of 99.2% with spectral centroid.
Ghouti et al. [47]	The authors propose a framework for robust identification of audio content by using short robust hashing codes, which applies the forward balanced multiwavelet (BMW) to transform each audio frame using 5 decomposition levels, and after the distribution of the subbands’ coefficients into 32 different blocks, the estimation quantization (EQ) scheme and the hashes are computed.
Cook et al. [48]	The authors propose a system that allows audio content identification and association of metadata in very restricted embedded environments. The authors report that the system has better performance than the method based on a more traditional n-dimensional hashing scheme, but it achieves results with 2% less accuracy.
Seo et al. [49]	The authors propose an audio fingerprinting method based on the normalized Spectral Subband Centroid (SSC), where the match is performed using the square of the Euclidean distance. The normalized SSC obtains better results than the widely-used features, such as tonality and Mel Frequency Cepstral Coefficients (MFCC).
Haitsma et al. [50]	The authors present an approach to audio fingerprinting, but it has negligible effects on other aspects, such as robustness and reliability. They proved that the developed method is robust in case of linear speed changes.
Haitsma et al. [51]	The authors present an approach to audio fingerprinting, in which the fingerprint extraction is based on the extraction of a 32-bit sub-fingerprint every 11.8 millis. They also develop a fingerprint database and implement a two-phase search algorithm, achieving an excellent performance, and allowing the analytical modeling of false acceptance rates.

**Table 3:** Distribution of the features extracted in the studies.

Features	Average Accuracy of Features	Number of Studies
Fast Fourier Transform (FFT)	93.85%	16
Thresholding	90.49%	6
Normalized spectral subband centroid (SSC)	93.44%	5
Mel-frequency cepstrum coefficients (MFCC)	97.30%	4
Maximum	87.57%	3
Local peaks and landmarks	82.32%	3
Shannon entropy	99.10%	2
Rényi entropy	99.10%	2
MPEG-7 descriptors	97.50%	2
Spectral bandwidth	97.10%	2
Spectral flatness measure	97.10%	2
Modified discrete cosine transform (MDCT)	93.00%	2
Constant Q transform (CQT)	85.40%	2
Short-time Fourier transform (STFT)	84.50%	2
Average	83.00%	2
Minimum	83.00%	2
Sum of the spectrum modulus of every frame	100.00%	1
Sum of global spectrum modulus in two stages	100.00%	1
Local energy centroid (LEC)	100.00%	1
Time-frequency power spectral	100.00%	1
Long-term logarithmic modified discrete cosine transform (DCT) modulation coefficients (LMDCT-MC)	99.67%	1
Bit packing	99.20%	1
Spectral band energy	99.20%	1
Spectral crest factor	99.20%	1
Spectral similarity	99.00%	1
Timbral texture	99.00%	1
Band periodicity	99.00%	1
Linear prediction coefficient derived cepstral coefficients (lpccs)	99.00%	1
Zero crossing rate	99.00%	1
Octaves	99.00%	1
Single local description	96.00%	1
Multiple local description	96.00%	1
Chroma vectors	96.00%	1
MP3 signatures	96.00%	1
Time averaging	96.00%	1
Quadratic interpolation	96.00%	1
Sinusoidal quantification	96.00%	1
Frequency-axial discretization	96.00%	1
Time-axial warping	96.00%	1
Logarithmic moduled complex lapped transform spectral peaks	86.50%	1
Correlation coefficient	70.00%	1
Matching score	70.00%	1

**Table 4:** Distribution of the methods implemented in the studies.

Methods	Average Accuracy of Methods	Number of Studies
Other methods	90.78%	15
Two level search algorithm	99.84%	2
Likelihood estimation	97.10%	3
Principal Component Analysis (PCA)	90.13%	2
Hamming distances between each fingerprint	83.50%	2
Streaming audio fingerprinting (SAF)	100.00%	1
Human auditory system (HAS)	100.00%	1
Divide and locate (DAL)	100.00%	1
Gaussian mixture models (GMM) modelling	99.20%	1
Local discriminant bases (LDBS)-based automated multigroup audio classification system	99.00%	1
Linear discriminant analysis (LDA)	99.00%	1
Local maximum chroma energy (LMCE)	99.00%	1
Expanded hash table lookup method	97.40%	1
Query Context (QUC)-tree	96.00%	1
Improved Real-Time TV-channel Recognition (IRTR)	95.00%	1
Sub-Nyquist fudio fingerprinting system	93.43%	1
Logarithmic moduled complex lapped transform (LMCLT) peak pair	86.50%	1
TO-Combo-SAD (Threshold Optimized Combo SAD) algorithm	84.25%	1
Support vector machine (SVM)	84.25%	1
Hough Transform between each fingerprint	81.00%	1
Masked audio spectral keypoints (MASK)	58.00%	1

**Table 5:** Potential accuracies for the top most accurate methods vs. top most mean accurate features (mean accuracies equal or higher than 99%, according to its authors).

		SAF	HAS	DAL	TLS	GMM	LDBS	LDA	LMCE
		***100.00%***	***100.00%***	***100.00%***	***99.84%***	***99.20%***	***99.00%***	***99.00%***	***99.00%***
Local energy centroid (LEC)	***100.00%***	100.00%	100.00%	100.00%	99.92%	99.60%	99.50%	99.50%	99.50%
Sum of global spectrum modulus in two stages	***100.00%***	100.00%	100.00%	100.00%	99.92%	99.60%	99.50%	99.50%	99.50%
Sum of the spectrum modulus of every frame	***100.00%***	100.00%	100.00%	100.00%	99.92%	99.60%	99.50%	99.50%	99.50%
Time-frequency power spectral	***100.00%***	100.00%	100.00%	100.00%	99.92%	99.60%	99.50%	99.50%	99.50%
Long-term logarithmic modified discrete cosine transform (DCT) modulation coefficients (LMDCT-MC)	***99.67%***	99.84%	99.84%	99.84%	99.76%	99.44%	99.34%	99.34%	99.34%
Bit packing	***99.20%***	99.60%	99.60%	99.60%	99.52%	99.20%	99.10%	99.10%	99.10%
Spectral band energy	***99.20%***	99.60%	99.60%	99.60%	99.52%	99.20%	99.10%	99.10%	99.10%
Spectral crest factor	***99.20%***	99.60%	99.60%	99.60%	99.52%	99.20%	99.10%	99.10%	99.10%
Rényi entropy	***99.10%***	99.55%	99.55%	99.55%	99.47%	99.15%	99.05%	99.05%	99.05%
Shannon entropy	***99.10%***	99.55%	99.55%	99.55%	99.47%	99.15%	99.05%	99.05%	99.05%
Band periodicity	***99.00%***	99.50%	99.50%	99.50%	99.42%	99.10%	99.00%	99.00%	99.00%
Linear prediction coefficient derived cepstral coefficients (lpccs)	***99.00%***	99.50%	99.50%	99.50%	99.42%	99.10%	99.00%	99.00%	99.00%
Octaves	***99.00%***	99.50%	99.50%	99.50%	99.42%	99.10%	99.00%	99.00%	99.00%
Spectral similarity	***99.00%***	99.50%	99.50%	99.50%	99.42%	99.10%	99.00%	99.00%	99.00%
Timbral texture	***99.00%***	99.50%	99.50%	99.50%	99.42%	99.10%	99.00%	99.00%	99.00%
Zero crossing rate	***99.00%***	99.50%	99.50%	99.50%	99.42%	99.10%	99.00%	99.00%	99.00%
